# Correction: van Doorn et al. Deriving Motor States and Mobility Metrics from Gamified Augmented Reality Rehabilitation Exercises in People with Parkinson’s Disease. *Sensors* 2025, *25*, 7172

**DOI:** 10.3390/s26041155

**Published:** 2026-02-11

**Authors:** Pieter F. van Doorn, Edward Nyman, Koen Wishaupt, Marjolein M. van der Krogt, Melvyn Roerdink

**Affiliations:** 1 Department of Human Movement Sciences, Faculty of Behavioural and Movement Sciences, Vrije Universiteit Amsterdam, Amsterdam Movement Sciences, 1081 BT Amsterdam, The Netherlands; 2 Department of Nutrition and Movement Sciences, NUTRIM Institute of Nutrition and Translational Research in Metabolism, Faculty of Health, Medicine and Life Sciences, Maastricht University, 6211 LK Maastricht, The Netherlands; 3 Department of Nutrition and Movement Sciences, MHeNs Institute of Mental Health and Neurosciences, Faculty of Health, Medicine and Life Sciences, Maastricht University, 6211 LK Maastricht, The Netherlands; 4 Department of Data and AI, Strolll Ltd., Stafford ST16 2LP, UK; 5 Department of Rehabilitation Medicine, Amsterdam UMC Location Vrije Universiteit Amsterdam, 1081 HV Amsterdam, The Netherlands; 6 Rehabilitation & Development, Amsterdam Movement Sciences, 1081 HV Amsterdam, The Netherlands; 7 Department of Science, Strolll Ltd., Stafford ST16 2LP, UK

**Text Correction**

An error was identified in the original publication [1]. A correction has been made to remove the erroneous pitch correction applied to the augmented reality–derived vertical position, as originally described in Section 2.5.1 and applied to derive gait parameters in Section 2.5.1. and squat parameters in Section 2.5.3. This removal has slightly affected the gait and squat metrics reported in Section 3.3 and Table 5 and in the abstract. In addition, the parts in Sections 4.1.3 and 4.2 of the Discussion, in which the pitch correction was addressed, have been removed. 

**Error in Table**

As a consequence of removing the erroneous pitch correction, the results presented in Table 5 of the original publication were incorrect. The corrected data are provided below. The authors state that the scientific conclusions remain unaffected. This correction was approved by the Academic Editor, and the original publication has been updated accordingly.

**Table 5:** Concurrent validity statistics for absolute agreement in mobility metrics between systems.

Motor State and Mobility Metric	Mean ± SD (SEM)	Mean ± SD (SEM)	Bias (95% Limits of Agreement)	*t*-Statistics or Wilcoxon Signed-Rank-Statistics ^1^	ICC_(A,1)_
**Straight walking**	**AR**	**Theia3D head**			
Step length (m)	0.61 ± 0.11 (0.03)	0.60 ± 0.11 (0.03)	−0.01 (−0.09 0.08)	t(14) = 0.71, *p* = 0.489	0.938
Max. gait speed (m/s)	1.66 ± 0.35 (0.01)	1.66 ± 0.35 (0.01)	0.00 (−0.03 0.03)	t(14) = −0.62, *p* = 0.543	0.999
Cadence (steps/min)	118.59 ± 14.04 (5.44)	120.37 ± 14.58 (5.65)	1.79 (−13.70 17.27)	t(14) = −0.88, *p* = 0.396	0.850
**Turning**	**AR**	**Theia3D pelvis**			
Turn duration (s)	2.05 ± 0.22 (0.13)	2.07 ± 0.20 (0.09)	0.02 (−0.17 0.20)	t(14) = −0.69, *p* = 0.504	0.904
Peak angular velocity (deg/s)	136.98 ± 16.95 (4.24)	116.06 ± 18.24 (4.54)	−20.92 (−42.34 0.51)	t(14) = 7.41, ***p* < 0.001**	0.477
**Squatting**	**AR**	**Theia3D head**			
Squat duration (s)	2.12 ± 0.57 (0.04)	2.11 ± 0.58 (0.04)	−0.01 (−0.12 0.10)	t(14) = 0.82, *p* = 0.425	0.995
Squat depth (m)	0.49 ± 0.18 (0.03)	0.45 ± 0.16 (0.03)	−0.03 (−0.08 0.02)	t(14) = 4.75, ***p* < 0.001**	0.972
**Transfers**	**AR**	**Theia3D head**			
Sit-to-stand duration (s)	1.20 ± 0.20 (0.03)	1.21 ± 0.17 (0.03)	0.00 (−0.07 0.08)	t(14) = −0.44, *p* = 0.664	0.979
Stand-to-sit duration (s)	1.57 ± 0.38 (0.06)	1.59 ± 0.41 (0.06)	0.01 (−0.15 0.18)	t(14) = −0.67, *p* = 0.513	0.978

^1^ Significant *p*-values in bold.
