# Supplementary material for: Deriving Motor States and Mobility Metrics from Gamified Augmented Reality Rehabilitation Exercises in People with Parkinson’s Disease
Source: Sensors (Basel). 2025 Nov 24;25(23):7172. doi: 10.3390/s25237172 (PMC12694056; doi:10.3390/s25237172)
Supplement: Supplementary file 1 [file sensors-25-07172-s001.zip › sensors-3959151-supplementary.pdf]

Supplementary Material S1. Strolll AR gamified exercises

**Game**	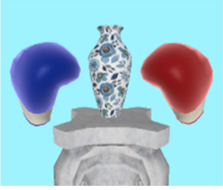<br>**Smash!**	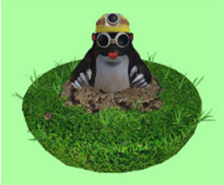<br>**Mole Patrolll**	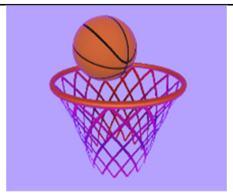<br>**Basketball**	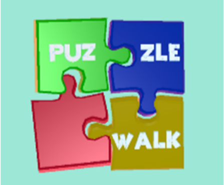<br>**Puzzle Walk**	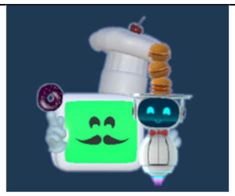<br>**Wobbly Waiter**
**Description of the exercise**	A boxing rehabilitation exercise to train gait, dynamic balance, weight shifting and turning.<br><br>The goal is to smash as many items as possible from two plinths as they appear, demanding alternate left and right punches to promote weight shifting, with available items alternating between the plinths to promote walking and turning.	A goal-directed walking rehabilitation exercise to train gait initiation, walking adaptability, dynamic balance, turning, stopping and strength (when performed in squat mode).<br><br>The goal is to stomp as many moles as possible by scanning the room, spotting where they appear, and stomping on them either with both feet or squatting on them (a game-mode setting) before they disappear.	A sit-to-stand rehabilitation exercise to train dynamic balance and lower-limb muscle strength.<br><br>The goal is to score as many points as possible by completing sit-to-stand or squat-to-stand movements (a game-mode setting) to spawn a set of three basketballs, and throw them into the hoop.	A goal-directed walking rehabilitation exercise to train gait, dynamic balance, turning, stopping and functional reaching.<br><br>The goal is to find puzzle pieces in the room, pick them up by reaching and grabbing them with your hand and then placing them on the easel to complete the puzzle before the time runs out.	A cued rehabilitation exercise focusing on walking at a set speed, turning and standing-up/sitting-down up from/in a chair, with an element of cognitive challenge while seated (memory retention).<br><br>The goal is to memorize a cafe order from a customer, assemble the order by selecting the buttons with the correct food items in the right sequence, and deliver the order to the customer's table within a prescribed amount of time, as cued by the waiter's speed.
**Exercise settings (level)**	• Difficulty level (number of required punches before the items drop from the plinth: 10)<br>• Distance between the plinths (3 meters)	• Difficulty level (time before mole disappears, 60 seconds per mole)<br>• Game mode (stomp mode)	• Difficulty level (number of required squats per three balls: 3)<br>• Game mode (squat mode)<br>• Rhythmic music (off)	• Difficulty level (number of puzzle pieces: 48)<br>• Game mode (puzzle piece height: high, hip, knee, floor)	• Difficulty Level (number of items to remember: 3)<br>• 10-meter walk test (10 sec.)<br>• Timed Up-and-Go test (10 sec.)<br>• Gait speed adjustment: 0%)
